# Judgements of a speaker’s personality are correlated across differing content and stimulus type

**DOI:** 10.1371/journal.pone.0204991

**Published:** 2018-10-04

**Authors:** Gaby Mahrholz, Pascal Belin, Phil McAleer

**Affiliations:** 1 School of Psychology, University of Glasgow, Glasgow, United Kingdom; 2 Institut des Neurosciences de la Timone, UMR 7289, CNRS and Université Aix-Marseille, Marseille, France; University of Lleida, SPAIN

## Abstract

It has previously been shown that first impressions of a speaker’s personality, whether accurate or not, can be judged from short utterances of vowels and greetings, as well as from prolonged sentences and readings of complex paragraphs. From these studies, it is established that listeners’ judgements are highly consistent with one another, suggesting that different people judge personality traits in a similar fashion, with three key personality traits being related to measures of valence (associated with trustworthiness), dominance, and attractiveness. Yet, particularly in voice perception, limited research has established the reliability of such personality judgements across stimulus types of varying lengths. Here we investigate whether first impressions of trustworthiness, dominance, and attractiveness of novel speakers are related when a judgement is made on hearing both one word and one sentence from the same speaker. Secondly, we test whether what is said, thus adjusting content, influences the stability of personality ratings. 60 Scottish voices (30 females) were recorded reading two texts: one of ambiguous content and one with socially-relevant content. One word (~500 ms) and one sentence (~3000 ms) were extracted from each recording for each speaker. 181 participants (138 females) rated either male or female voices across both content conditions (ambiguous, socially-relevant) and both stimulus types (word, sentence) for one of the three personality traits (trustworthiness, dominance, attractiveness). Pearson correlations showed personality ratings between words and sentences were strongly correlated, with no significant influence of content. In short, when establishing an impression of a novel speaker, judgments of three key personality traits are highly related whether you hear one word or one sentence, irrespective of what they are saying. This finding is consistent with initial personality judgments serving as elucidators of approach or avoidance behaviour, without modulation by time or content. All data and sounds are available on OSF (osf.io/s3cxy).

## Introduction

First impressions play a fundamental role in life as they guide our thoughts, affect subsequent behaviours, and, in turn, influence decisions towards a person [1, 2]. The human voice is one of the main sources providing first impressions of a speaker’s identity, such as gender, race, age, and vocation [3–10], or physical attributes like height and weight, physical strength, or health and fertility [11–16]. Furthermore, largely based on non-verbal vocal information (such as pitch and intonation) rather than verbal content (i.e. what is said), it has been shown that rapid assessments are made about a speaker’s affective state [17–19], confidence level [20], perceived intelligence [21], and personality [1, 6, 22–24]. In turn, such rapid judgements impact our business decisions [25], voting and political preferences [26–31], whom we hire [21], whom we laugh with [19, 32], and whom we are attracted to [22–24]. Be them termed as thin-slice personality judgements or zero acquaintance judgements (e.g. [1, 33–37]), first impression judgements are formed rapidly, from little information, and show high consistency between raters [7, 38–42], suggesting that listeners perceive novel speakers in a largely similar fashion. For clarity, the term “first impressions” refers to brief (e.g. 100 ms) or prolonged exposure to a target (e.g. 5 minutes) where there is no interaction between participant and target [2, 43, 44], as opposed to what might be termed “first interactions” where participants interact together for a period before rating the other [45].

Furthermore, whilst a person can be rated on numerous personality traits, it has been shown that first impression judgements are predominantly established through a combination of two distinct personality traits: trustworthiness, and dominance [1, 46]. Principal component analysis of Likert ratings scales, conducted on faces, and subsequently replicated in voices, suggests a first component based on valence [38, 41], frequently aligned to traits of trustworthiness [1], integrity [27], or likeability [47], whereas a second component is commonly related to dominance [1, 38, 41, 47], or physical prowess [27]. However, whilst the two dimensional space is well established for faces and voices, Sutherland and colleagues [41], using ratings of ambient everyday images of faces, proposed a third component associated with youthfulness/attractiveness. Physical attractiveness has also previously been proposed to mediate first impression judgements from faces [48]. Overall, the dimensional space is proposed to have a social relevance as it reflects a person’s intent, via trustworthiness/valence judgements, and their ability to enact that intent, through dominance ratings [1, 38]. Grounding this theory within voices, this emphasises the importance of the non-verbal signals within a voice for conveying this information. Theoretically, it should not matter what someone says for you to make an informative judgment concerning their intent (see e.g. Puts et al. [49] for a discussion on how pitch and formants have been shaped by evolutionary pressures to enable the signalling of dominance across male anthropoids).

As mentioned, a prominent finding from the dimensional approach to personality judgements is that studies tend to show a high degree of consistency across ratings for the perceived personality of a speaker. This is found in both face and voice research, and is largely irrespective of the veracity of the judgements [2, 31, 50–52]. Further, in voice research, this cross-participant consistency has been established within given specific durations of vocalisations or utterances; high inter-rater reliability for ratings has been found using sub-second utterances of vowels or words [1, 22, 46, 53, 54], as well as from longer sentences and passages [6, 7, 27, 29, 30, 47]. For illustration, McAleer et al. [1] reported very high Cronbach’s Alpha for ratings towards voices across a number of personality traits (all alpha’s > .88) which is in line with the high inter-rater reliability found in similar face perception studies (all alphas > .9 in [38]; = .98 in [39]; > .7 in [41, 42]; > .86 in [55]) though with some variation depending on traits (e.g. attractiveness: .95 - .97, trustworthiness: .92, aggressiveness: .75 - .89 in [40]). Thus, within specific lengths of vocal stimuli presentations, ratings of novel speakers across listeners, are all similar in fashion.

Similarly, looking at reliability of personality traits ***across*** presentation durations, Willis and Todorov [40] found that ratings of trustworthiness, competence, likeability, aggressiveness, and attractiveness for faces, showed moderate to strong positive correlations after 100 ms, 500 ms, and 1000 ms, when compared to ratings made without time constraints. Only participants’ confidence in their own judgements increased as a function of duration. Likewise, again using photographs of faces, Bar et al. [33] reported medium positive correlations between ratings at 39 ms and 1700 ms. The authors indicated that the lower threshold was sufficient for reliable assessments of threat but not intelligence, supporting the theory that rapid first impressions serve as a mean of self-preservation and help determine appropriate approach-avoidance behaviour [1, 33, 38]. The idea being that it should not require much information to decide whether a stranger is friend or foe. Finally, Todorov and colleagues [39] obtained a similar finding, again for faces, showing 33 ms of exposure to be sufficient to distinguish between trustworthy- and untrustworthy-looking stimuli. Whilst correlations with control ratings improved between 33 ms and 100 ms, increased exposure duration did not significantly increase the correlations.

In voice research, though there are limited studies that consider the reliability of personality judgements across varying lengths of stimulus types, similar findings have been shown as in face research. Comparing trust ratings across different monophthong vowels (A, E, O), albeit with limited change in stimulus length, Rezlescu et al. [46] found strong positive correlations across recordings by the same speaker, suggesting a degree of stability of perceived personality within a speaker. This research suggests that judgements are driven largely by non-verbal cues and not speech content. Likewise, Ferdenzi and colleagues [56] found no significant effect of stimulus type (vowels, three-vowel combinations, word) on ratings of attractiveness. Furthermore, Ferdenzi et al. [56] also synthetically manipulated stimulus duration, as well as stimulus type, and found that the percentage by how much the stimulus was lengthened, decreased attractiveness ratings–i.e. a word lengthened by 88% would on average receive a lower score in attractiveness than a word only lengthened by 4%, suggesting that experimenter manipulations can influence ratings. However, given Rezlescu et al. [46] used vowel utterances of similar duration, whilst Ferdenzi and colleagues [56] utilised artificially shortened and lengthened stimuli, it remains to be established whether ratings of perceived personality in naturally occurring utterances of differing lengths, from the same speaker, remain similar or related. Furthermore, given that it is standard for ratings in personality studies to be obtained with different groups of listeners (cf. [56]), the reliability of personality ratings to the same speaker, across varying speech segment lengths (e.g. word vs. sentence), within the same listener is as yet unknown.

An additional variable for consideration when comparing speech and voices would be the content of what is actually said. Contrary to presenting a static face for a longer period of time [33, 39, 40], speech is dynamic and the semantic meaning and/or acoustics change with prolonged exposure, which could in turn affect perceived personality of the speaker by the listener [46, 57–59]. Previous voice research has used a variety of content, for example: monophthong vowel sounds, as a truly content-absent condition [22, 24, 46, 56, 60]; incoherent voices [57, 61, 62]; content-neutral words, i.e. numbers [63], the alphabet [64, 65], time of day [66]; emotional words [67]; words directed towards the listener, i.e. ‘hello’ or equivalents thereof [1, 23, 56, 62]; sentences directed towards the listener [29, 30, 68]; neutral sentences of limited content, e.g. the Rainbow Passage [27, 69, 70]; as well as longer passages [6, 47, 71, 72], and periods of free speech [7, 27, 73–75].

The interaction of non-verbal cues with speech content is a highly relevant question, as this reflects our everyday occurrences. Imhof [71], using three extended speech scenarios focussing on stereotyping (fixing a bike tube (male), baking a shortcake (female), and read addresses (neutral)) found that content influenced ratings on the Big Five traits. Neutral content resulted in people being perceived as being less extraverted, less open, and more conscientious, whereas female-stereotype content was associated with more emotional stability. However, much of the work considering speech content on personality has used manipulated utterances in order to control for potential variables of non-interest. For example, Tsantani et al. [62] compared normal and reversed voicings from the same speaker and showed content had no effect on overall pitch preference. Conversely, Jones et al. [24] found that male preferences for female high pitched voices, often rated as attractive [1, 76], was reduced by sentiment of what was said. Using low and high pitched versions of the same voice saying either “I really like you” (interested) or “I don’t really like you” (disinterested), they found that preference for high pitch was strongest for interested clauses than disinterested clauses. Both clauses still indicated an overall preference for the low pitch voices, however, suggesting that it is only the extent of this preference that is ameliorated. The effect was not found when voices were played backwards or when rated by female listeners, suggesting an interaction between the pitch, speech content and listener sex. O’Connor and colleagues [77] showed that female listeners preferred lower pitched voices when comparing voices manipulated in pitch (low vs. high) to represent low or high economic status. However, when voices signalled high economic status, preference was not influenced by pitch. Finally, O’Connor and Barclay [78], looking at the relationship of voice pitch on pro- or antisocial sentiments, found that pitch did not influence judgements of prosocial statements, but results did show an additive effect when low pitch voices were heard expressing anti-social sentiments, rating them most untrustworthy of all. Taken together, these studies would suggest that the content of the speech can influence personality judgements, however, the findings are perhaps offset by the relatively small sample of voices used (e.g. 4–6 voices), the manipulation to these voices [78], and/or the 2AFC comparison task [62]. As such, the question as to how pitch and content interact to establish a judgement of a personality remains open.

The current study, therefore, explores the reliability, or relatedness, of personality ratings from voices across two stimulus types (word vs. sentence) and two varying content conditions. Trustworthiness, dominance, and attractiveness were chosen as these are the key traits highlighted in a principal component analysis of personality ratings. To investigate the effects of varying speech segment lengths on ratings of perceived personality, word and sentence stimuli were extracted from emotionally neutral recordings of each speaker. To explore the influence of content, two content conditions were created; the content-ambiguous condition was designed as non-contextual to a listener, whereas the content-relevant condition would be socially relevant to the listener, specifically addressing the target, and purposely aimed at a student population given our likely sample (as in [29]). We would equate this contrast of content to face research, establishing perceived personality from faces looking directly at a participant (akin to our content-relevant stimuli) and faces looking or turned away from the participant (akin to our content-ambiguous stimuli) [41, 79]. Furthermore, age range was restricted to 17–30 years for speakers, as well as listeners, to minimise the effects of a potential age-related positivity bias frequently reported in memory [80, 81] and face perception research [82, 83]. Based on previous studies in face research showing good reliability of perceived personality ratings across varying durations [33, 39, 40], positive moderate to strong correlations were predicted between short and long vocalisations from the same speaker. Secondly, in accordance with Tsantani et al.’s [62] using reverse speech as a content-absent condition, and given their use of similar stimuli, it was expected that speech content would have no effect on the perceived personality ratings of trustworthiness, dominance, and attractiveness. Moderate to strong correlations across trait ratings towards stimuli types (word vs. sentence) and of varying content would be indicative of perceived personality having a purpose in self-preservation and in being involved in establishing appropriate approach-avoidance behaviours [1, 38, 51]. This suggests decisions being formed rapidly without conscious decision-making. In contrast, no relationship between the word/sentence condition by the same speaker would indicate that such personality judgements serve limited function as a means of establishing approach-avoidance behaviour, perhaps implying that higher level cognitive processes are involved [84, 85].

## Materials and methods

### Ethics

All procedures (recording and experimental) were approved by the University of Glasgow Ethics Committee, and are in accordance with the ethical standards of the 1964 Declaration of Helsinki. Given the online nature of the experiment, all experimental participants provided consent by pressing a confirmation button (“Yes”; the alternative option “No” did not allow participants to progress to the experiment) after reading on-screen statements acknowledging their participation would be voluntary, their data stored and treated anonymously, and that they could withdraw at any time. Additionally, participants in the voice recording part of the experiment gave written consent to their recording being made available as part of an open-access database for future experiments.

### Participants

#### Voice recording

60 native English speakers (30 females: 20.2 ± 2.95 years (range: 17–27 years); 30 males: 23.2 ± 3.75 years (range: 17–30 years)) were recruited for stimuli recording via the University of Glasgow School of Psychology Subject Pool. Advertising was placed for Scottish participants, between 17 and 30 years of age without speech impairments. All speakers were reimbursed for their contribution; either receiving £3, or the equivalent in participation credits as part of their Psychology undergraduate degree. The sample size of 30 voices per voice sex was determined using R (R Core Team (2017), Version 3.4.2) with RStudio (Version 1.0.143) and Pwr Package [86]) prior to commencing the experiment with a view of obtaining a power of 0.9 (lowest Pearson correlation coefficients from pilot data was ~ .55, based on a two-tailed α = .05).

#### Online rating experiment

181 new participants [138 female: 20.1 ± 2.45 years (range: 18–30 years); 43 males: 21.3 ± 2.78 years (range: 18–27 years)] took part in the online voice rating experiment. Participant recruitment was via the same means and criteria as for the voice recording participants, with the exception of not having participated in the voice recording stage. Incentives were equivalent to those given in the voice recording stage.

### Stimuli

The recordings took place in a custom-made sound-attenuated chamber, within the School of Psychology, University of Glasgow, using Audacity (.wav format, 16-bit mono, 44100 Hz; http://www.audacityteam.org/). 60 speakers were recorded individually reading two unfamiliar texts (see S1 Appendix) approximately 5 times. Participants were instructed to read the passages in a natural, emotionally neutral voice; without any instruction to convey a particular emotion. To form content-ambiguous stimuli, “colours” (stimulus type: word), and “Some have accepted it as a miracle without physical explanation” (stimulus type: sentence) were extracted from the Rainbow Passage excerpt [69]. For the content-relevant conditions “Hello” (stimulus type: word), and “I urge you to submit your essay by the end of the week” (stimulus type: sentence) were selected from a passage created for this study, which was tailored towards a student population (as in [29]). The Rainbow Passage excerpts (content-ambiguous stimuli) were chosen due to being of approximately similar word length to the respective content-relevant stimuli, avoided repeating words from the content-relevant condition where possible, and for the sentences to be comprehensible sentences free from pronouns that would suggest the phrases were directed at the listener; akin to face research using faces turned away from the perceiver or towards the perceiver [41, 79]. The most fluently spoken words and sentences were selected from the recordings of each speaker given that interruptions and disfluencies impact on perceived personality [87]. Stimuli were extracted via Audacity, and subsequently normalised for intensity through Matlab (The MathWorks, Inc., Natwick, Massachusetts, USA) as louder voices are perceived as more dominant [87]. See Table 1 for average stimuli duration and standard deviations, and OSF depository (osf.io/s3cxy) and Supplementary Information for auditory stimuli (S1 Stimuli) and acoustic data (S2 Dataset). In regards to actual time durations, although of approximately similar word length, content-ambiguous stimuli were significantly longer than content-relevant stimuli in both voice sexes and stimulus types (all t’s > 2.6, all p’s < .05).

**Table 1:** Average stimuli duration per content condition and stimulus type.

Content Condition	Stimulus Type	Female Voices		Male Voices	
		Average Duration (ms)	Standard Deviation (ms)	Average Duration (ms)	Standard Deviation (ms)
Ambiguous	Word	470.0	61.8	451.6	58.1
Ambiguous	Sentence	3172.8	277.3	3019.6	264.2
Relevant	Word	394.6	49.0	411.1	60.1
Relevant	Sentence	2362.4	179.6	2313.8	203.5

### Procedure

The experiment was conducted online through the Experiment webpages of the School of Psychology, University of Glasgow (http://experiments.psy.gla.ac.uk/). Participants were instructed to complete the experiment in a quiet environment, through headphones or speakers. Participants were randomly assigned to one of three personality traits (trustworthiness, dominance, or attractiveness) for either female or male voices (see Table 2) and were instructed to rate each stimulus using a visual analogue scale (VAS) slider ranging from “not at all [trait]” (left) to “extremely [trait]” (right). For their respective personality trait and sex of stimuli voice, each participant was presented with 4 blocks of stimuli (ambiguous words, ambiguous sentences, relevant words, and relevant sentences) in a counterbalanced order of four possibilities changing only one variable between blocks at a time to prolong the naivety of the participants as regards the overall purpose of the study: 1. Ambiguous word, Ambiguous sentence, Relevant sentence, Relevant word, 2. Ambiguous sentence, Ambiguous word, Relevant word, Relevant sentence; 3. Relevant word, Relevant sentence, Ambiguous sentence, Ambiguous word; 4. Relevant sentence, Relevant word, Ambiguous word, Ambiguous sentence. Within each block, each of the 30 voice stimuli of that block (e.g. female speaker 1 saying “Hello” in the relevant word block) was presented twice, resulting in a total of 240 ratings per participant. Untimed breaks were given between each block with the experiment lasting approximately 30 minutes per participant.

**Table 2:** Number of female and male participants separated by personality trait and voice sex.

Personality Trait	Voice Sex	Number of Female Participants	Number of Male Participants	Total number of participants
Trustworthiness	Female	22	8	30
Trustworthiness	Male	24	7	31
Dominance	Female	23	7	30
Dominance	Male	24	6	30
Attractiveness	Female	22	8	30
Attractiveness	Male	23	7	30

### Data analysis

Given the online nature of the experiment, and to remove participants responding arbitrarily, pre-stipulated exclusion criterion similar to [1] stated that for each participant 2/3 of all the second ratings of the stimuli should fall within 1 standard deviation of the first ratings. For that, each participant’s ratings were transformed into z-scores, and the percentage of difference larger than 1 SD between 1^st^ and 2^nd^ rating determined. No participants were excluded for violating this criterion.

A series of Welch’s t-tests revealed no significant differences between the overall ratings of male and female participants across all traits (see Table 2 above; Female Voices: t_trustworthiness_ (57.997) = 1.187, p = .240; t_dominance_ (57.365) = -0.414, p = .680; t_attractiveness_ (57.840) = -1.963, p = .054; Male Voices: t_trustworthiness_ (56.429) = -1.879, p = .065; t_dominance_ (55.565) = -0.497, p = .621; t_attractiveness_ (51.820) = 1.963, p = .125). Bruckert et al. [88] as well as previous pilot studies from our lab have also shown no differences in perceived personality between male and female listeners. However, all analyses were conducted regardless of sex of listener given the small number of male listeners in each group. Further, all analyses were conducted at the item level (i.e. an individual voice) whereby for each voice, an average score was calculated from the mean of the original VAS ratings of each participant, for that voice. All raw data (original rating data for first and second ratings of all participants) is available with the manuscript (S1 Dataset) or on the OSF depository (osf.io/s3cxy).

## Results

### Inter-rater reliability across participants

Cronbach’s alpha was calculated to establish a level of a measure of inter-rater reliability between listeners within a given condition. Overall, results revealed a high level of inter-rater reliability (all alphas > .86; see Table A in S1 File for breakdown by condition).

### Comparison of personality traits by stimulus type (word vs. sentence)

**Fig 1:**
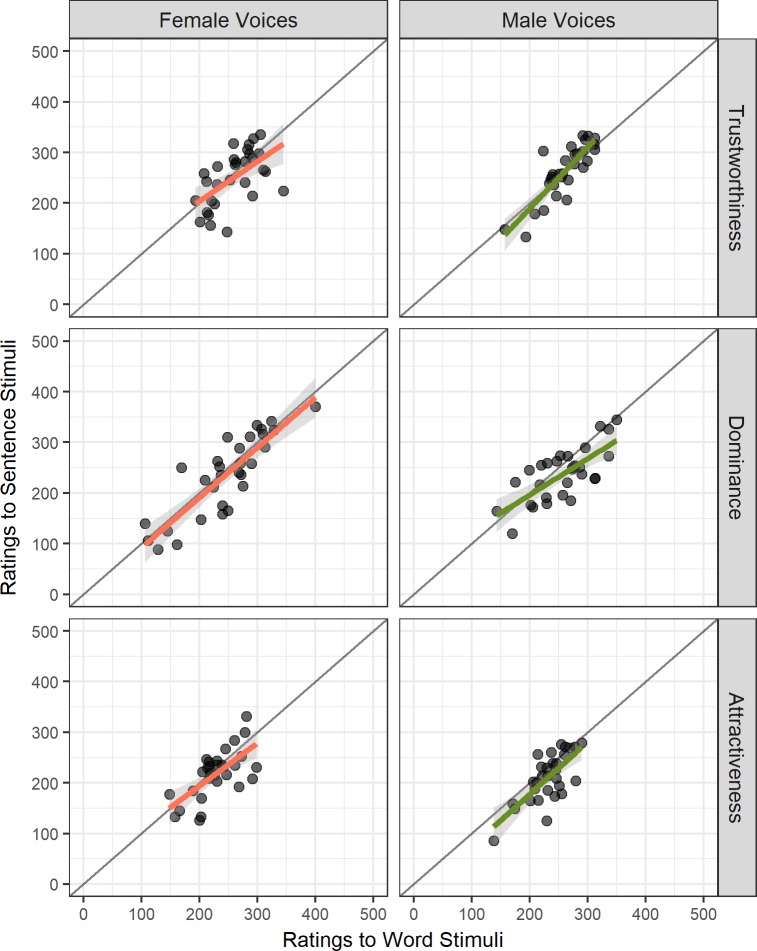
**Scatterplot of VAS ratings for words versus sentences in female and male voices for trustworthiness (top), dominance (middle), and attractiveness (bottom panel).** Female Voices (left) and regression slope (Orange); Male Voices (right) and regression slope (Green); each dot represents a single voice; grey line represents r = 1.

Pearson correlation coefficients were calculated testing the relationships between personality trait ratings of words versus sentences within the same speaker for the traits of trustworthiness, dominance, and attractiveness (between variable). All tests revealed positive moderate to strong linear relationships (see Fig 1; Female Voices: r_trustworthiness_ = .578, p < .001; r_dominance_ = .857, p < .001; r_attractiveness_ = .672, p < .001; Male Voices: r_trustworthiness_ = .846, p < .001; r_dominance_ = .729, p < .001; r_attractiveness_ = .721, p < .001).

On further inspection of the data, five outliers within either the sentences or words conditions were identified based on boxplot analysis using 1.5 times the Inter-Quartile Range away from the 25^th^ and 75^th^ quartiles of the data. Pearson correlation coefficients were subsequently obtained on both the original and the outlier-removed data sets, and Fisher's r-z transformed correlations for the comparison of correlation values showed no significant difference between the Pearson correlation values of the full sample versus those obtained from the subset with outliers removed (see Table B in S1 File; all absolute z differences < 1.96). Therefore, no voices were excluded from the data set as outliers, and all were used in further analyses.

### Linear mixed effect model: Stimulus type by content

**Fig 2:**
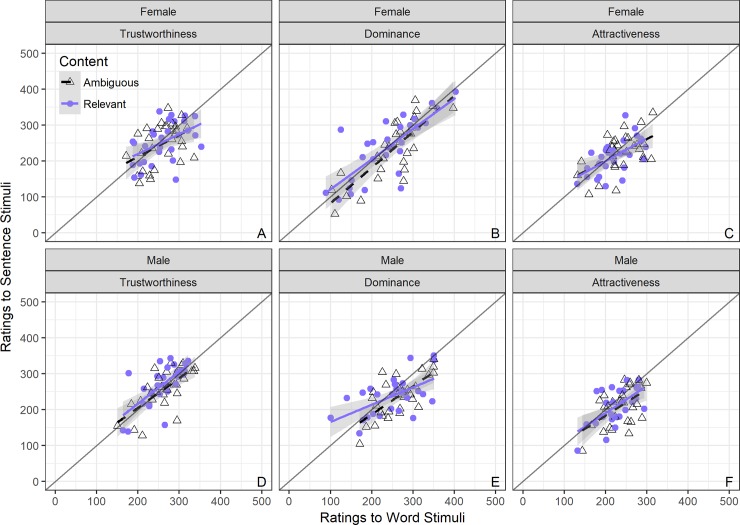
**Scatterplots of VAS ratings for words versus sentences by content, in female (top) and male voices (bottom panels) for trustworthiness (left), dominance (middle), and attractiveness (right panel).** Content-ambiguous (black dashed regression slope; open triangles represent individual voices) versus Content-relevant (blue solid regression slope; closed circles represent individual voices); grey line represents r = 1.

To further address the question of whether ratings of perceived personality are related when participants hear one word compared to one sentence, and how this is influenced by Content, we fitted a series of Linear Mixed Effects Models with random intercepts specified for each participant and each voice [89, 90]. As our intent is to look within sex and within traits, and not between sex or between trait, models were fitted separately for male and female stimuli and for each personality trait rated. The dependent variable in the models were personality ratings to sentence stimuli. This order was chosen as previous research [1] had used one-word stimuli and therefore we looked at predicting personality ratings upon hearing sentences from ratings upon hearing words. Random slopes by-participant and by-voice (i.e. by-item) were fitted for the two content conditions (deviation coded with content-relevant = -.5 and content-ambiguous = .5). Fixed effects were specified for personality ratings to one word stimuli and for content variable. The full relationships and model estimates can be seen in Fig 2 and Tables C-E in S1 File.

#### Trustworthiness

For both female and male voices (Fig 2 Panels A & D) the models showed a significant positive effect of stimulus type word on sentence (**Females**: beta = .291, 95CI [0.244, 0.339], p < .001; **Males**: beta = .352, 95CI [0.304, 0.4], p < .001). No other effects were found to be significant for female voices (all ps > .62) or male voices (all ps > .75). The models and visualisations suggest that ratings of trustworthiness for words and sentences are significantly correlated and that they are more positive when rating voices from a single word than when rating voices from a full sentence. Overall, the relationship between trustworthiness ratings when hearing one word versus hearing one sentence were all moderate to strong regardless of content.

#### Dominance

Again, for both female and male voices (Fig 2 Panels B & E), the models showed a significant positive effect of stimulus type word (**Females**: beta = .210, 95CI [0.156, 0.257], p < .001; **Males**: beta = .234, 95CI [0.185, 0.282], p < .001). No other effects were found to be significant for female voices (all ps > .05) nor male voices (all ps > .05). The models and visualisations suggest that ratings of dominance for words and sentences are significantly correlated and that they are more positive when rating voices from a single word than when rating voices from a full sentence. Overall, the relationship between dominance ratings when hearing one word versus hearing one sentence were all moderate to strong regardless of content.

#### Attractiveness

Finally, and as in the two previous traits, for both female and male voices (Fig 2 Panels C & F) the models showed a significant positive effect of stimulus type word on sentence (**Females**: beta = .269, 95CI [0.219, 0.32], p < .001; **Males**: beta = .322, 95CI [0.273, 0.373], p < .001). No other effects were found to be significant for female voices (all ps > .25) nor male voices (all ps > .12). The models and visualisations suggest that ratings of attractiveness for words and sentences are significantly correlated and that they are more positive when rating voices from a single word than when rating voices from a full sentence. Overall, the relationship between attractiveness ratings when hearing one word versus hearing one sentence were all moderate to strong regardless of content.

### Comparison of personality traits by content

**Fig 3:**
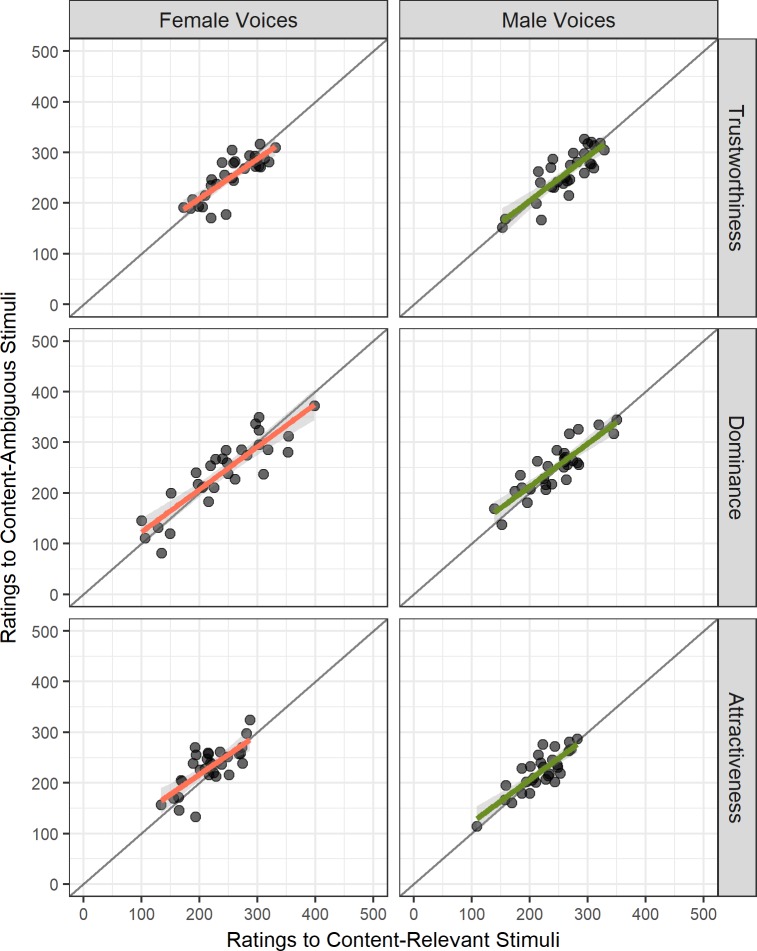
**Scatterplot of VAS ratings for content-relevant versus content-ambiguous in female and male voices for trustworthiness (top), dominance (middle) and attractiveness (bottom panel).** Female Voices (left) and regression slope (Orange); Male Voices (right) and regression slope (Green); each dot represents a single voice; grey line represents r = 1.

Pearson correlation coefficients were calculated to test the relationships between ratings of content-ambiguous versus content-relevant stimuli within the same speaker (separately for the personality traits of trustworthiness, dominance, and attractiveness). All tests revealed positive moderate to strong linear relationships (see Fig 3; Female Voices: r_trustworthiness_ = .821, p < .001; r_dominance_ = .883, p < .001; r_attractiveness_ = .742, p < .001; Male Voices: r_trustworthiness_ = .831, p < .001; r_dominance_ = .870, p < .001; r_attractiveness_ = .834, p < .001).

Further analysis identified seven outliers within either the ambiguous or relevant content dimensions using the same procedure as before. Pearson correlation coefficients were obtained on the outlier-removed data set. Fisher's r-z transformed correlations were subsequently computed for the comparison of correlation values and showed no significant difference between the Pearson correlation values of the original data set versus those obtained from the outlier-removed subset (see Table F in S1 File; all absolute z differences < 1.96). Therefore, again, no voices were excluded from the data set as outliers, and all were used in further analyses.

### Linear mixed effect models: Content by stimulus type

**Fig 4:**
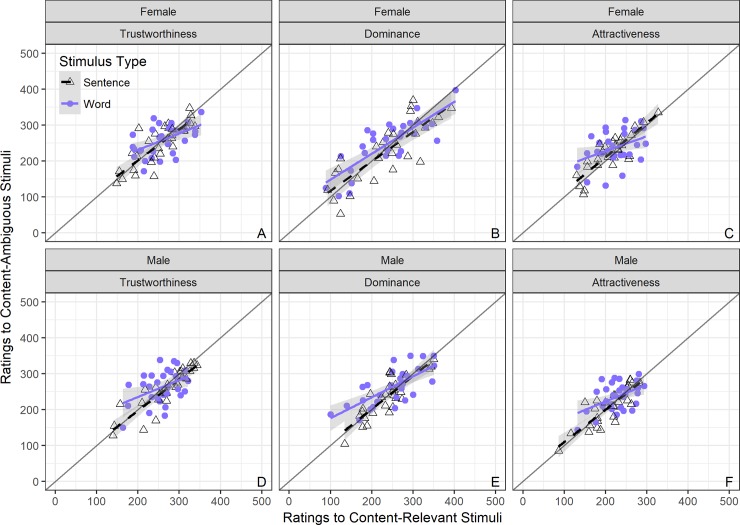
**Scatterplots of VAS ratings for content-relevant versus content-ambiguous by stimulus type (word vs. sentence), in female (top) and male voices (bottom panels) for trustworthiness (left), dominance (middle), and attractiveness (right panel).** Sentences (black dashed regression slope; open triangles represent individual voices) versus Words (blue solid regression slope; closed circles represent individual voices); grey line represents r = 1.

As above, to address the question of whether ratings of perceived personality are related when participants hear speech with content relevant to them (i.e. content intended to be directed towards them) compared to ambiguous content (i.e. not intended to be directed towards them), and how this is influenced by stimulus type (word vs. sentence), we fitted a series of Linear Mixed Effects Models with random intercepts specified for each participant and each voice. Again, models were fitted separately for male and female stimuli and for each personality trait rated. The dependent variable in the models were personality ratings to the content-ambiguous stimuli; this order was chosen again to follow McAleer and colleagues [1] who had previously used relevant stimuli (i.e. “Hello”). Random slopes by-participant and by-voice (i.e. by-item) were fitted for the two stimulus types (deviation coded as word = -.5 and sentence = .5). Fixed effects were specified for personality ratings to content-relevant ratings and for the length of stimulus variable. The full relationships and model estimates can be seen in Fig 4 and Tables G-I in S1 File.

#### Trustworthiness

In regards to trustworthiness ratings, for female and male voices (Fig 4 Panels A & D) the model showed a significant positive effect of relevant content condition (**Females:** beta = .329, 95CI [0.286, 0.374], p < .001; **Males:** beta = .247, 95CI [0.204, 0.291], p < .001), a main effect of stimulus type (**Females:** beta = -37.259, 95CI [-66.705, -8.365], p < .05; **Males:** beta = -80.515, 95CI [-108.182, -53.23], p < .001), and an interaction between content and stimulus type (**Females:** beta = .112, 95CI [0.025, 0.2], p < .01; **Males:** beta = .275, 95CI [0.191, 0.359], p < .001). The interaction was resolved by fitting LMEs for predicting ratings to content-ambiguous stimuli from content-relevant stimuli separately for words and then for sentences. Both models fitted random intercept models only for participant and voice and showed a positive effect of content type (***word only***—**Females:** beta = .275, 95CI [0.211, 0.34], p < .001; **Males:** beta = .123, 95CI [0.063, 0.183], p < .001; ***sentence only***—**Females:** beta = .400, 95CI [0.34, 0.461], p < .001; **Males:** beta = .404, 95CI [0.344, 0.466], p < .001). The models and visualisations suggest that trustworthiness ratings between content-relevant and content-ambiguous stimuli are significantly correlated, and are generally overall more positive in the relevant than the ambiguous content condition. The interaction would suggest that relevant sentences are significantly better than relevant words at predicting ambiguous content. In general, comparing ratings for content-ambiguous to content-relevant stimuli, all relationships appear moderate to strong, but significantly stronger in sentences than in words.

#### Dominance

In regards to dominance ratings, for female and male voices (Fig 4 Panels B & E) the model showed a significant positive effect of relevant content condition (**Females:** beta = .214, 95CI [0.17, 0.259], p < .001; **Males:** beta = .360, 95CI [0.318, 0.403], p < .001), a main effect of stimulus type (**Females:** beta = -35.973, 95CI [-63.884, -7.906], p < .05; **Males:** beta = -76.15, 95CI [-102.789, -49.696], p < .001), and an interaction between content and stimulus type in male voices only (**Females:** beta = .075, 95CI [-0.012, 0.161], p = .07; **Males:** beta = .219, 95CI [0.136, 0.301], p = .001). The interaction in male voices was resolved by fitting a similar LME as in trustworthiness. Both word and sentence models in male voices showed a positive effect of content type (***word only***—**Males:** beta = .264, 95CI [0.204, 0.326], p < .001; ***sentence only***—**Males:** beta = .487, 95CI [0.429, 0.546], p < .001). The models and visualisations suggest that dominance ratings between content-relevant and content-ambiguous stimuli are significantly correlated, and are generally higher overall in the ambiguous but more positive than the relevant content condition. The interaction in male voices would suggest that relevant sentences are significantly better than relevant words at predicting ambiguous content. In general, comparing ratings for content-ambiguous to content-relevant stimuli, all relationships appear moderate to strong, but significantly stronger in sentences than in words.

#### Attractiveness

Finally, in regards to attractiveness ratings, for female and male voices (Fig 4 Panels C & F) the model showed a significant positive effect of relevant content condition (**Females:** beta = .297, 95CI [0.252, 0.342], p < .001; **Males:** beta = .318, 95CI [0.278, 0.358], p < .001), a main effect of stimulus type (**Females:** beta = -43.886, 95CI [-71.116, -16.722], p < .01; **Males:** beta = -70.42, 95CI [-94.309, -46.565], p < .001), and an interaction between content and stimulus type (**Females:** beta = .152, 95CI [0.064, 0.241], p < .001; **Males:** beta = .225, 95CI [0.149, 0.303], p < .001). The interaction was resolved as previously in trustworthiness. Both models fitted random intercept models only for participant and voice and showed a positive effect of content type (***word only***—**Females:** beta = .229, 95CI [0.164, 0.294], p < .001; **Males:** beta = .217, 95CI [0.159, 0.275], p < .001; ***sentence only***—**Females:** beta = .383, 95CI [0.322, 0.445], p < .001; **Males:** beta = .447, 95CI [0.394, 0.502], p < .001). As in trustworthiness and dominance, the models and visualisations suggest that attractiveness ratings between content-relevant and content-ambiguous stimuli are significantly correlated, and are generally higher overall in the ambiguous but more positive than the relevant content condition. The interaction would suggest that relevant sentences are significantly better than relevant words at predicting ambiguous content. In general, comparing ratings for content-ambiguous to content-relevant stimuli, all relationships appear moderate to strong, but significantly stronger in sentences than in words.

## Discussion

The purpose of the current study was to assess how changes to both the stimulus type (word vs. sentence) and content of an utterance impacts on the relatedness (or reliability) of perceived personality traits, such as trustworthiness, dominance, and attractiveness, for a novel speaker. As a first pass measure of inter-rater reliability, high Cronbach alpha values were obtained indicating participants showed strong agreement across their judgements within a given condition and within personality traits. This is in alignment with previous literature [38–42]. Secondly, moderate to strong correlations were found between ratings of the same speaker saying one word and saying a full sentence, for both voice sex, in each of the tested personality traits. However, this effect was noticeably stronger in male voices than in female voices. Finally, when comparing perceived personality ratings on hearing socially-relevant content versus ambiguous content, correlations were again moderate to strong for all three key personality traits, with no obvious differences across voice sex. Linear mixed effects modelling revealed that trait ratings for sentences and socially-ambiguous content can be significantly predicted from words and socially-relevant content respectively. However, ratings to words and content-relevant stimuli were generally more positive compared to sentences and content-ambiguous stimuli respectively, and that correlations, i.e. the reliability of personality ratings, were stronger for when rating sentences than for words.

Expanding on these results in turn, the high inter-rater reliability (i.e. through Cronbach alpha) for trustworthy, dominant, and attractive words and sentences, suggests a strong degree of similarity between listeners’ perceived personality ratings of speakers, and is in agreement with previous face and voice literature [1, 38–42, 46, 64]. For example, McAleer et al. [1] reported Cronbach’s alpha of similar strength to the current study, implying that listeners not only make judgements about a speaker after just one word, but that these judgements are agreed across listeners. Our findings strengthen results from McAleer and colleagues [1] suggesting that 500 ms of exposure is sufficient to make trait inferences from an unfamiliar voice. By extension, the current findings indicate that listeners also largely agree on what a trustworthy, dominant, or attractive voice sounds like after only 3 seconds of exposure to that voice. All in all, the high inter-rater reliability values from the current study, aligned with those previously reported within the literature, may suggest a form of prototypical coding similar to that established for voice identity [60], whereby listeners make their judgement in regards to an internalised normative representation. Indeed, Ponsot et al. [91] highlighted normative pitch contours of vocal trustworthiness and dominance using reverse correlation, though further work is required to determine the true generalisability of these representations across stimuli, speaker, and listener [92, 93].

In regards to stimulus type (word vs. sentence), our findings suggest that ratings of the perceived personality of a novel speaker are highly similar across two relatively short exposure times which is in line with studies using face stimuli [39, 40]. Shown here now in voices implies that an initial assessment of how trustworthy, dominant, or attractive a speaker sounds, assessed after hearing a short exposure to their voice, would be similar to the same judgement made after a longer duration. A theoretical explanation for these similarities of judgements between words and sentences is proposed via Oosterhof and Todorov’s [38] 2D model of face evaluation, suggesting that an initial judgement of valence/trust aims to establish a person’s intent, whereas the dominance judgement establishes the ability for that person to carry out their intent. McAleer et al. [1] proposed a similar evaluation system in voices which is aimed at self-preservation, again assessing whether a person’s intentions are harmful or not. Extending the model to attractiveness makes sense if we consider mate selection as part of self-preservation, and potentially supports the inclusion of attractiveness as a key trait [41, 48]. Furthermore, our results showing that ratings for sentences were higher than for words, across all three traits though more so for attractiveness and trustworthiness than dominance, support previous findings for faces [39, 40]. It is possible that this difference was weakest in dominance as previous literature has shown this trait to be driven by more stable voice metrics, such as formant and HNR, whereas trust and attractiveness may be more related to pitch [1, 49, 70, 73]. Also, audio-visual integration research suggests that dominance is more driven by the voice, whereas trustworthiness and attractiveness appear driven either by the face or the integration of modalities [46, 55]. Thus, perceived dominance in voices may be so prevalent that it does not matter whether you hear one word or one sentence. An alternative explanation may be in consideration of a false positive, akin to the smoke-detector principle [94]: assessing someone as non-trustworthy/-dominant/-attractive when indeed they are. A poor judgement may not have severe consequences when establishing trustworthiness or attractiveness, but might prove detrimental for self-preservation when making assessments of dominance, given a proposed association between dominance, physical strength, and fighting ability [16, 95–97]. Future work utilising social game theory and established consequences of decisions would help to elaborate on the differences between judgements of traits across various speech segment lengths.

An additional finding on the correlations based on stimulus type (word vs. sentence) was that the strengths of the correlations were notably stronger for male voices than for female voices; only dominance showed comparable strengths across the two sexes. Again, that dominance should be strongest and most similar in both sexes may again be due to the underlying acoustics (e.g. formant dispersion) not changing across utterances, whereas the variability of trust and attractiveness is perhaps more related to the variability of pitch and intonation [1, 95–97]. Alternatively, the difference may lie in the demographic make-up of our sample. There is an abundance of psychological research whereby the samples are predominantly female (see [76] for discussion). The case applies here with approximately a two to one ratio female to male, though balanced across all traits and conditions. As such, this difference may be the result of one sex agreeing more on the ratings of the opposite sex or agreeing more on ratings of their own sex, when it comes to judgements of trustworthiness and attractiveness. Previous studies, such as Jones et al. [24], show clear differences between how the two sexes rate these traits or make preferential judgements on these traits, and whilst no strong conclusion can be drawn from this study, it poses an interesting avenue for further development using a more balanced sample in regards to sex.

When considering content, our findings support the notion that the perceived personality of a male or female speaker will be reliable across varying utterances regardless of what is said. The more positive judgements to socially relevant stimuli perhaps reflect that speech content is personally directed to the speaker, akin to a person facing you as opposed to away from you [98, 99]. This is in agreement with findings by Tsantani et al. [62] who showed no significant differences in regards to a general preference for high and low pitched voices, when using socially-relevant words and their temporally-reversed form. Here, we look to extend the findings to the key personality traits of trustworthiness, dominance, and attractiveness in more natural speech patterns. Conversely however, Imhof [71] reported an effect of content on perceived personality judgements of the Big Five personality traits. Likewise, experiments using a 2AFC comparison task of high and low pitched voices have reported effects of content for traits such as trustworthiness and attractiveness [24, 76–78]. Differences between studies may simply lie in the design [62]. Alternatively, we may find that the relatedness of personality judgements from one situation to the next is a function of longer durations than those tested here (30 seconds, a minute or longer) or of degree of interaction, after which reassessment of the speaker can take place based on additional information. In the current study, the average duration of the sentence stimuli was approximately 3 seconds whereas Imhof’s [71] speech segments were between 20–30 seconds. Herein may be the distinction between “first impression” judgements based on brief exposure, and an established view of a person’s character which Satchell [45] may refer to as judgements after “first interaction”. For example, you initially perceive a person speaking in your periphery as threatening, and this judgement is the same for durations up to a certain timeframe (for example 10 seconds) but given prolonged exposure or the ability to converse with them, you realise they were telling a joke and reassess them as friendly. Within the current study, at a minimum, we show that within the first 3 seconds of exposure to a female or male voice, content does not influence the perceptions of trustworthiness, dominance, or attractiveness to the extent that the perceived personality varies greatly. The point at which reassessment of a perceived personality takes place remains an open question.

Continuing this point, whilst we have shown ratings across differing stimulus types and contents are relatively reliable, what we cannot yet conclude with the current paradigm is how the perception of personality actually develops over time; whether the first word we hear determines our percept and we seek confirmation of this percept through further exposure (i.e. we use information solely to vindicate our initial percept), or whether we are continually updating our percept as we listen longer to the same voice. Future experiments employing finer temporal-gating paradigms [39, 40, 100], novel continual response paradigms (e.g. keypressing paradigms in [101, 102]) or some derivative of event segmentation [103] would do well to investigate this point further.

Finally, in consideration of generalisability [104], whilst the current findings are informative, we should consider potential limitations in an attempt to ground the work, and not overreach its application beyond acknowledging the use of a WEIRD sample from a deliberately restricted age range [105]. One merit of the work is that we used a sample of voices larger than that more commonly found [24, 62, 76–78] and whilst this is a step in the right direction, it is still short of complementary work in face perception where stimuli count can be in the hundreds [41, 106]. As such, it is yet unclear how strong the effects would be in a larger sample (though power was high for our correlations) or across cultures [107]. Secondly, it has been noted that changing the task in personality studies may lead to contrasting findings [62], and research would benefit from a direct comparison of methods, both in terms of response (see study 1 vs. study 2 in [78]), and in terms of temporal gating of stimuli (see [40], and [33] vs. current study). In addition to this, obtaining responses from the same participant is highly insightful, but responses are potentially convolved with participants’ memory of previous ratings as opposed to actual perception. Whilst we cannot rule this out in the current study, we would suggest that memory of previous ratings does not play a major factor here, given both the reasons previously stated [108, 109], the volume of stimuli and conditions, and the consistent responses to the personality trait. Finally, we must consider that the utterances we used are from an infinite pool of possible human speech, which can vary on a range of metrics such as duration and order of words. For example, in our stimuli the word “hello” was a phrase in itself, whereas “colours” was the final word in a longer sentence (see S1 Appendix). Given that vocal acoustics vary across duration and position within an utterance [57], the selection of the two words for the stimuli may have contributed to higher variability within words, as compared to sentences. Thus, we cannot negate the findings of previous studies concluding that content has influence on perception of personality [24, 62, 76–78], as other utterances, controlled for elements such as duration or valence of content, may give differing results to the current findings. That said, and despite these limitations mentioned, the study still showed moderate to strong relationships between the conditions across all three personality traits, indicating that a speaker’s voice does carry certain non-verbal information that would lead to their personality being perceived in a similar fashion across differing situations.

In summary, it is proposed that rapid judgements of trustworthiness, dominance, and attractiveness are consistent across listeners, and reliable across short durations of varying content. This finding holds true for male as well as female voices and we propose this to be driven by a self-preservation purpose, serving as elucidator of approach or avoidance behaviour. The results of this study strengthen and expand our understanding of trait judgements from voices, and further highlight the similarities between the processing of voices and faces in regards to perceiving the personality of another.

## Supporting information

S1 Appendix Voice recording texts and instructions. (DOCX)

S1 File Tables A to I. (DOCX)

S1 Dataset Raw data. (CSV)

S2 Dataset Acoustic data. (CSV)

S1 Stimuli Zip Folder of stimuli. (ZIP)
