# Supplementary material for: Judgements of a speaker’s personality are correlated across differing content and stimulus type
Source: PLoS One. 2018 Oct 4;13(10):e0204991. doi: 10.1371/journal.pone.0204991 (PMC6171871; doi:10.1371/journal.pone.0204991)
Supplement: S1 Appendix — (DOCX) [file pone.0204991.s001.docx]

S1 Appendix. Voice recording texts and instructions.

Bold highlighting was added to emphasize which words and sentences were used as stimuli in the online rating experiment. No highlighting was used in the voice recording stage of the experiment.

Please read both of the texts 5 times each in an emotionally neutral voice.

***Text 1: Excerpt from the Rainbow Passage [62]***

When the sunlight strikes raindrops in the air, they act like a prism and form a rainbow. The rainbow is a division of white light into many beautiful **colors**. These take the shape of a long, round arch, with its path high above and its two ends apparently beyond the horizon. There is, according to legend, a boiling pot of gold at one end. People look, but no one ever finds it. When a man looks for something beyond his reach, his friends say he is looking for the pot of gold at the end of the rainbow.

Throughout the centuries men have explained the rainbow in various ways. **Some have accepted it as a miracle without physical explanation.** To the Hebrews it was a token that there would be no more universal floods.

***Text 2: Telephone scenario (as in [29])***

As I sat in my room, suddenly the phone rang. ‘**Hello**’, said the voice. ‘This is your lecturer. **I urge you to submit your essay by the end of the week.**’ What a surprise it was to receive such a call on a Sunday night!
