# Supplementary material for: Judgements of a speaker’s personality are correlated across differing content and stimulus type
Source: PLoS One. 2018 Oct 4;13(10):e0204991. doi: 10.1371/journal.pone.0204991 (PMC6171871; doi:10.1371/journal.pone.0204991)
Supplement: S1 File — (DOCX) [file pone.0204991.s002.docx]

S1 File

Table A: Cronbach’s alpha scores, as a measure of inter-rater reliability, for female and male voices, per content, stimulus type, and personality trait.

| Personality Trait | Content Condition | Stimulus Type | Cronbach's alpha for Female Voices | Cronbach's alpha for Male Voices |
| --- | --- | --- | --- | --- |
| Trustworthiness | Total | | .94 | .95 |
| Trustworthiness | All Word | | .92 | .92 |
| Trustworthiness | All Sentence | | .95 | .94 |
| Trustworthiness | All Ambiguous | | .92 | .94 |
| Trustworthiness | All Relevant | | .92 | .94 |
| Trustworthiness | Ambiguous | Word | .88 | .91 |
| Trustworthiness | Ambiguous | Sentence | .93 | .92 |
| Trustworthiness | Relevant | Word | .91 | .89 |
| Trustworthiness | Relevant | Sentence | .92 | .92 |
| Dominance | Total | | .99 | .95 |
| Dominance | All Word | | .98 | .95 |
| Dominance | All Sentence | | .98 | .93 |
| Dominance | All Ambiguous | | .98 | .94 |
| Dominance | All Relevant | | .98 | .93 |
| Dominance | Ambiguous | Word | .97 | .91 |
| Dominance | Ambiguous | Sentence | .97 | .92 |
| Dominance | Relevant | Word | .97 | .93 |
| Dominance | Relevant | Sentence | .97 | .89 |
| Attractiveness | Total | | .95 | .93 |
| Attractiveness | All Word | | .93 | .89 |
| Attractiveness | All Sentence | | .94 | .93 |
| Attractiveness | All Ambiguous | | .94 | .93 |
| Attractiveness | All Relevant | | .93 | .90 |
| Attractiveness | Ambiguous | Word | .92 | .89 |
| Attractiveness | Ambiguous | Sentence | .93 | .92 |
| Attractiveness | Relevant | Word | .92 | .86 |
| Attractiveness | Relevant | Sentence | .91 | .89 |

Table B: Comparison of Pearson correlation coefficients of original data set with a subset without outliers on the stimulus type dimension.

| Personality Trait | Voice Sex | Pearson r for original data set | N1 | Pearson r for outlier- removed data set | N2 | Zr1 | Zr2 | z |
| --- | --- | --- | --- | --- | --- | --- | --- | --- |
| Trustworthiness | Female | .578 | 30 | .578 | 30 | 0.66 | 0.66 | 0.00 |
| Dominance | Female | .857 | 30 | .857 | 30 | 1.28 | 1.28 | 0.00 |
| Attractiveness | Female | .672 | 30 | .645 | 28 | 0.81 | 0.77 | 0.17 |
| Trustworthiness | Male | .846 | 30 | .768 | 28 | 1.24 | 1.02 | 0.82 |
| Dominance | Male | .729 | 30 | .729 | 30 | 0.93 | 0.93 | 0.00 |
| Attractiveness | Male | .721 | 30 | .623 | 29 | 0.91 | 0.73 | 0.65 |

N1 & N2, number of stimuli for data sets 1 & 2; Zr1 & Zr2, Fisher’s r-z transformed correlations for samples 1 & 2; z, absolute value needs to be greater than 1.96 (assuming a 2 -tailed test and alpha = 0.05) for the null hypothesis to be rejected

Table C: Full results of models testing for effects on trustworthiness ratings between hearing a single word and a full sentence from the same speaker, by content condition. DV in the model was trustworthiness ratings to a sentence, with random slopes by-participant and by-voice fitted for the two content conditions (deviation coded: relevant content = -.5 and ambiguous content = .5). Fixed effects were specified for trustworthiness ratings to one word stimuli and for Content. Separate models were fitted for Male and Female voice stimuli.

| **Female Trustworthiness** | | | | | |
| --- | --- | --- | --- | --- | --- |
| Fixed Effects | Estimate | Std. Error | df | t-value | p-value |
| Intercept | 174.68 | 12.383 | 87.5 | 14.106 | <2e-16 |
| Word | 0.291 | 0.024 | 1777.9 | 12.102 | <2e-16 |
| Content | 0.880 | 13.96 | 240.5 | 0.063 | .950 |
| Word:Content | -0.023 | 0.046 | 1204.3 | -0.500 | .617 |
|  | | | | | |
| **Male Trustworthiness** | | | | | |
| Intercept | 201.735 | 11.313 | 82.9 | 17.833 | <2e-16 |
| Word | 0.234 | 0.024 | 1709.3 | 9.765 | <2e-16 |
| Content | -26.085 | 13.393 | 280.8 | -1.948 | .053 |
| Word:Content | 0.053 | 0.046 | 1040.7 | 1.157 | .247 |

Table D: Full results of models testing for effects on dominance ratings between hearing a single word and a full sentence from the same speaker, by content condition. DV in the model was dominance ratings to a sentence, with random slopes by-participant and by-voice fitted for the two content conditions (deviation coded: relevant content = -.5 and ambiguous content = .5). Fixed effects were specified for dominance ratings to one word stimuli and for Content. Separate models were fitted for Male and Female voice stimuli.

| **Female Dominance** | | | | | |
| --- | --- | --- | --- | --- | --- |
| Fixed Effects | Estimate | Std. Error | df | t-value | p-value |
| Intercept | 184.82 | 14.628 | 52.80 | 12.634 | <2e-16 |
| Word | 0.206 | 0.025 | 1745.3 | 8.090 | <2e-15 |
| Content | -30.435 | 15.475 | 137.3 | -1.967 | .051 |
| Word:Content | 0.084 | 0.047 | 907.6 | 1.782 | .075 |
|  | | | | | |
| **Male Dominance** | | | | | |
| Intercept | 147 | 10.88 | 98.80 | 13.515 | <2e-16 |
| Word | 0.352 | 0.024 | 1735 | 14.715 | <2e-16 |
| Content | -4.392 | 13.73 | 183.7 | -0.320 | .749 |
| Word:Content | -0.006 | 0.044 | 819.7 | -0.146 | .884 |

Table E: Full results of models testing for effects on attractiveness ratings between hearing a single word and a full sentence from the same speaker, by content condition. DV in the model was attractiveness ratings to a sentence, with random slopes by-participant and by-voice fitted for the two content conditions (deviation coded: relevant content = -.5 and ambiguous content = .5). Fixed effects were specified for attractiveness ratings to one word stimuli and for Content. Separate models were fitted for Male and Female voice stimuli.

| **Female Attractiveness** | | | | | |
| --- | --- | --- | --- | --- | --- |
| Fixed Effects | Estimate | Std. Error | df | t-value | p-value |
| Intercept | 156.882 | 10.205 | 84.0 | 15.368 | <2e-16 |
| Word | 0.269 | 0.025 | 1560.7 | 10.710 | <2e-16 |
| Content | 14.588 | 12.784 | 226.7 | 1.141 | .255 |
| Word:Content | -0.037 | 0.048 | 896.6 | -.0776 | .438 |
|  | | | | | |
| **Male Attractiveness** | | | | | |
| Intercept | 135.91 | 11.538 | 89.9 | 11.779 | <2e-16 |
| Word | 0.322 | 0.025 | 1765 | 12.989 | <2e-16 |
| Content | 12.620 | 12.847 | 192.7 | 0.982 | .327 |
| Word:Content | -0.072 | 0.046 | 833.5 | -1.563 | .118 |

Table F: Comparison of Pearson correlation coefficients of original data set with a subset without outliers on the content dimension.

| Personality Trait | Voice Sex | Pearson r for original data set | N1 | Pearson r for outlier- removed data set | N2 | Zr1 | Zr2 | z |
| --- | --- | --- | --- | --- | --- | --- | --- | --- |
| Trustworthiness | Female | .821 | 30 | .821 | 30 | 1.16 | 1.16 | 0.00 |
| Dominance | Female | .883 | 30 | .883 | 29 | 1.39 | 1.39 | 0.01 |
| Attractiveness | Female | .742 | 30 | .727 | 27 | 0.95 | 0.92 | 0.11 |
| Trustworthiness | Male | .831 | 30 | .788 | 29 | 1.19 | 1.07 | 0.45 |
| Dominance | Male | .870 | 30 | .848 | 29 | 1.33 | 1.25 | 0.30 |
| Attractiveness | Male | .834 | 30 | .772 | 29 | 1.20 | 1.03 | 0.64 |

N1 & N2, number of stimuli for data sets 1 & 2; Zr1 & Zr2, Fisher’s r-z transformed correlations for samples 1 & 2; z, absolute value needs to be greater than 1.96 (assuming a 2 -tailed test and alpha = 0.05) for the null hypothesis to be rejected

Table G: Full results of models testing for effects on trustworthiness ratings between hearing a relevant content and ambiguous content from the same speaker, by stimulus type. DV in the model was trustworthiness ratings of ambiguous content, with random slopes by-participant and by-voice fitted for the two stimulus types (deviation coded: word = -.5 and sentence = .5). Fixed effects were specified for trustworthiness ratings to relevant content stimuli and for stimulus type. Separate models were fitted for Male and Female voice stimuli. Interaction effects were further explored by fitting LMEs looking at predicting ratings to ambiguous content from relevant content separately for words and then for sentences, with random intercepts fitted by-participant and by-voice, and fixed effects fitted for trustworthiness ratings to relevant stimuli.

| **Female Trustworthiness** | | | | | |
| --- | --- | --- | --- | --- | --- |
| Fixed Effects | Estimate | Std. Error | df | t-value | p-value |
| Intercept | 168.68 | 9.272 | 115.5 | 18.192 | <2e-16 |
| Relevant | 0.330 | 0.022 | 1750.3 | 14.969 | <2e-16 |
| Stimulus Type | -37.529 | 14.629 | 158.2 | -2.565 | .011 |
| Rel:StimType | 0.112 | 0.043 | 1502.2 | 2.591 | .010 |
| **Female Trustworthiness: Amb ~ Rel – Words Only** | | | | | |
| Intercept | 187.07 | 11.810 | 153.7 | 15.844 | <2e-16 |
| Relevant | 0.275 | 0.032 | 885.6 | 8.486 | <2e-16 |
| **Female Trustworthiness: Amb ~ Rel – Sentences Only** | | | | | |
| Intercept | 146.431 | 11.847 | 110.7 | 12.36 | <2e-16 |
| Relevant | .400 | 0.300 | 888.5 | 13.32 | <2e-16 |
|  | | | | | |
| **Male Trustworthiness** | | | | | |
| Intercept | 194.02 | 9.387 | 96.4 | 20.669 | <2e-16 |
| Relevant | 0.247 | 0.021 | 1802.2 | 11.533 | <2e-16 |
| Stimulus Type | -80.515 | 13.785 | 207.1 | -5.841 | <2e-08 |
| Rel:StimType | 0.274 | 0.042 | 1412.6 | 6.602 | <6e-11 |
| **Male Trustworthiness: Amb ~ Rel – Words Only** | | | | | |
| Intercept | 230.956 | 12.377 | 117.4 | 18.659 | <2e-16 |
| Relevant | 0.123 | 0.030 | 926.1 | 4.062 | 5.3e-05 |
| **Male Trustworthiness: Amb ~ Rel – Sentences Only** | | | | | |
| Intercept | 148.63 | 10.823 | 130.3 | 13.73 | <2e-16 |
| Relevant | 0.404 | 0.030 | 838.0 | 13.47 | <2e-16 |

Table H: Full results of models testing for effects on dominance ratings between hearing a relevant content and ambiguous content from the same speaker, by stimulus type. DV in the model was dominance ratings of ambiguous content, with random slopes by-participant and by-voice fitted for the two stimulus types (deviation coded: word = -.5 and sentence = .5). Fixed effects were specified for dominance ratings to relevant content stimuli and for stimulus type. Separate models were fitted for Male and Female voice stimuli. Interaction effects were further explored by fitting LMEs looking at predicting ratings to ambiguous content from relevant content separately for words and then for sentences, with random intercepts fitted by-participant and by-voice, and fixed effects fitted for dominance ratings to relevant stimuli.

| **Female Dominance** | | | | | |
| --- | --- | --- | --- | --- | --- |
| Fixed Effects | Estimate | Std. Error | df | t-value | p-value |
| Intercept | 189.327 | 12.961 | 54.1 | 14.918 | <2e-16 |
| Relevant | 0.214 | 0.022 | 1768.1 | 9.701 | <2e-16 |
| Stimulus Type | -38.973 | 13.840 | 123.8 | -2.599 | .0105 |
| Rel:StimType | 0.075 | 0.041 | 875.3 | 1.804 | .0715 |
|  | | | | | |
| **Male Dominance** | | | | | |
| Intercept | 163.078 | 9.724 | 92.4 | 16.771 | <2e-16 |
| Relevant | 0.360 | 0.021 | 1745.4 | 17.053 | <2e-16 |
| Stimulus Type | -76.150 | 13.286 | 157.3 | -5.732 | <5e-08 |
| Rel:StimType | 0.219 | 0.041 | 1232.2 | 5.393 | <9e-08 |
| **Male Dominance: Amb ~ Rel – Words Only** | | | | | |
| Intercept | 197.782 | 12.489 | 108.7 | 15.836 | <2e-16 |
| Relevant | 0.264 | 0.030 | 870.4 | 8.725 | <2e-16 |
| **Male Dominance: Amb ~ Rel – Sentences Only** | | | | | |
| Intercept | 120.960 | 10.978 | 110.2 | 11.02 | <2e-16 |
| Relevant | 0.486 | 0.029 | 885.0 | 16.68 | <2e-16 |

Table I: Full results of models testing for effects on attractiveness ratings between hearing a relevant content and ambiguous content from the same speaker, by stimulus type. DV in the model was attractiveness ratings of ambiguous content, with random slopes by-participant and by-voice fitted for the two stimulus types (deviation coded: word = -.5 and sentence = .5). Fixed effects were specified for attractiveness ratings to relevant content stimuli and for stimulus type. Separate models were fitted for Male and Female voice stimuli. Interaction effects were further explored by fitting LMEs looking at predicting ratings to ambiguous content from relevant content separately for words and then for sentences, with random intercepts fitted by-participant and by-voice, and fixed effects fitted for attractiveness ratings to relevant stimuli.

| **Female Attractiveness** | | | | | |
| --- | --- | --- | --- | --- | --- |
| Fixed Effects | Estimate | Std. Error | df | t-value | p-value |
| Intercept | 165.959 | 8.964 | 91.5 | 18.515 | <2e-16 |
| Relevant | 0.297 | 0.023 | 1780.9 | 13.094 | <2e-16 |
| Stimulus Type | -43.886 | 13.759 | 146.7 | -3.190 | <.002 |
| Rel:StimType | .152 | 0.045 | 1611.8 | 3.422 | <7e-04 |
| **Female Attractiveness: Amb ~ Rel – Words Only** | | | | | |
| Intercept | 186.063 | 12.013 | 109.0 | 15.489 | <2e-16 |
| Relevant | 0.229 | 0.033 | 897.3 | 6.945 | <8e-12 |
| **Female Attractiveness: Amb ~ Rel – Sentences Only** | | | | | |
| Intercept | 141.9 | 10.463 | 102.9 | 13.56 | <2e-16 |
| Relevant | 0.383 | 0.031 | 895.6 | 12.51 | <2e-16 |
|  | | | | | |
| **Male Attractiveness** | | | | | |
| Intercept | 154.09 | 8.870 | 84.1 | 17.371 | <2e-16 |
| Relevant | 0.318 | 0.020 | 1772.7 | 16.073 | <2e-16 |
| Stimulus Type | -70.42 | 11.928 | 124.2 | -5.904 | <4e-08 |
| Rel:StimType | 0.225 | 0.038 | 1092.4 | 5.989 | <3e-09 |
| **Male Attractiveness: Amb ~ Rel – Words Only** | | | | | |
| Intercept | 186.677 | 11.140 | 104.7 | 16.758 | <2e-16 |
| Relevant | 0.217 | 0.029 | 892.2 | 7.533 | <2e-13 |
| **Male Attractiveness: Amb ~ Rel – Words Only** | | | | | |
| Intercept | 115.394 | 10.152 | 89.2 | 11.37 | <2e-16 |
| Relevant | 0.448 | 0.027 | 878.7 | 16.75 | <2e-16 |
